# Salinity as a Key Factor on the Benthic Fauna Diversity in the Coastal Lakes

**DOI:** 10.3390/ani11113039

**Published:** 2021-10-23

**Authors:** Natalia Mrozińska, Katarzyna Glińska-Lewczuk, Krystian Obolewski

**Affiliations:** 1 Department of Hydrobiology, University of Kazimierz Wielki, 85-090 Bydgoszcz, Poland; mrozinska.natalia@ukw.edu.pl; 2 Department of Water Resources and Climatology, University of Warmia and Mazury, 10-719 Olsztyn, Poland; kaga@uwm.edu.pl

**Keywords:** macroinvertebrate communities, diversity, hydrological connectivity, brackish sea, ecotone zone

## Abstract

**Simple Summary:**

Salinity is a stress factor for benthic invertebrates. Based on a 2-year study of 9 coastal lakes along the southern Baltic Sea, representing freshwater, transitional, and brackish ecosystems, we have shown that benthic fauna was structured by sea water intrusion (=fluctuation of salinity). The increase in salinity gradient resulted in a decreasing trend in the richness and abundance of benthic species, while the diversity showed a slightly positive trend, but below statistical significance (*p* < 0.05). The abundance of benthic organisms was the highest in brackish costal lakes, where the marine component of fauna was identified. Due to the greatest instability of environmental conditions in lakes periodically linked with the sea (transitional), we found the lowest species number, α-diversity, and abundance, regardless of the season. Salinity appears as a key factor in controlling the functioning of the ecotone (coastal lakes, lagoons) between the marine and terrestrial environments. Salinity is a prerequisite for the proper assessment of the ecological status of coastal lakes and the development of effective methods of protecting coastal lakes.

**Abstract:**

Benthic communities were studied in nine Polish coastal lakes of the Baltic Sea; representing three levels of hydrological connection with the sea (isolated, periodically connected, and permanently connected), with resultant differences in salinity (freshwater, transitional, and brackish). The lakes classified in this way allowed us to investigate biodiversity in relation to the degree of environmental pressure. Stress intensity in coastal water bodies, resulting from contrasting marine and terrestrial influences, varied from mild to severe. Spatial variation in environmental predictors affected species richness more strongly than seasonal fluctuations. The broader the spatial salinity gradient, the smaller the species number recorded. Differences in the intensity of natural instability only slightly affected species number and α-diversity. In Baltic coastal lakes, characterized by low salinity (max. 7.5 PSU), benthic faunal communities were dominated by large populations of opportunistic species. This applied primarily to closed systems and those periodically influenced by seawater intrusion. The marine component of fauna played a more important role in increasing the diversity of benthos in permanently open water bodies (brackish). The highest density of benthic fauna was recorded in them, whereas low values were associated with the strongest instability, observed in lakes periodically linked with the sea (transitional).

## 1. Introduction

Coastal water bodies (lakes, drowned river valleys, and smaller intermittently open and closed lagoons) are sites of contact between marine and terrestrial environments, associated with mixing of variable amounts of fresh water from tributaries and seawater caused by the intrusion. As a result, their biotope is characterized by complex environmental gradients, comprising salinity, water exchange, morphometric features, nutrients, turbidity, and sediment properties [1,2]. Simultaneously, contributions of individual physicochemical predictors depend on the main hydrodynamic energy source [3,4,5]. In the coastal lakes with major tributaries (rivers), environmental gradients are determined by the amount of fresh water. By contrast, in the water bodies permanently connected with the sea, the gradients are affected mostly by the degree of seawater intrusion. In this way, two alternative stable states develop, with contrasting levels of water salinity [6,7]. According to the theory of alternative stable states, the system can remain in one of the possible states defined by a specific composition of biocenoses and habitat properties in ecologically significant time scales [8,9]. Specific cases are transitional lakes, with a periodic variable dominance of river water (freshwater-brackish) or seawater (brackish-freshwater), where fluctuations in environmental conditions are particularly high [10,11]. This can be also observed in river deltas [12,13]. This leads to continuous structural and functional changes within individual biocenoses colonizing the lakes, according to the assumptions of adaptive cycles [14,15].

The Baltic Sea is not a typical, salty sea. The mean salinity of its surface waters in the southern zone is only 7 PSU (range 2–20 PSU), i.e., 1/5 as high as in oceans. This is due to the dominance of the supply of fresh water (from rivers and precipitation) over the saltwater intrusion from Kattegat. As a result, its coastal lakes are characterized by a relatively small gradient of salinity, from <0.5 PSU to 7.5 PSU [16]. However, studies have shown that these ecosystems are highly unstable, leading to a remarkable variation in animal diversity [17,18]. The repeated pattern of species richness, decreasing from the places affected by seawater intrusion to internal parts of lakes, is well-documented [17,19,20,21]. Moreover, species diversity strongly depends on the possibility of migration [22] and varies in relation to increasing environmental instability caused by frequent changes in proportions between fresh and brackish waters [23]. In coastal ecosystems with limited seawater intrusion, some specialized animal species can tolerate environmental extremes and potentially develop large populations within a broad range of salinity levels [24]. Bamber et al. [25] found that eurytopic (opportunistic) species colonizing such ecosystems are better adapted to environmental variation, most probably because of the level of genetic plasticity, but under extreme salinity conditions, species richness is expected to decline [26]. It is necessary to investigate and compare the spatial and spatiotemporal patterns of fauna and flora in coastal lakes with various levels of natural stress to distinguish and quantify the major factors that structure communities, i.e., critical extremes and instability level as a predictor of species distribution [27].

Previous studies on benthic invertebrates inhabiting the Baltic coastal lakes have provided only some of the necessary information, as they were based only on information from selected lakes [17,21]. This study attempts to identify relations between the level of variation in abiotic conditions and fluctuations in the structure of invertebrate fauna of all southern Baltic coastal lakes along a gradient of salinity. The analyses concerned spatial and seasonal variation in benthic invertebrate communities (zoobenthos) in three types of coastal lakes: brackish (>3.0 PSU), transitional (0.5–3.0 PSU), and freshwater (<0.5 PSU). In this respect, it is the first comprehensive study of the taxonomic diversity of the benthic fauna of the lakes along the coast of the southern Baltic Sea.

## 2. Materials and Methods

**Figure 1:**
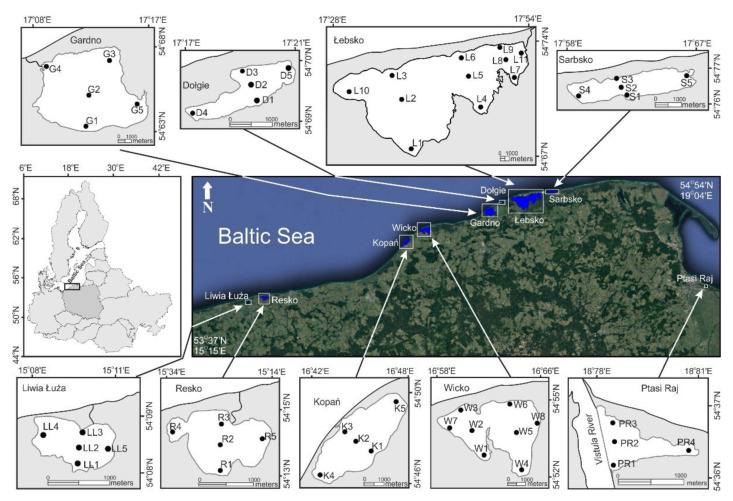
The location of sampling sites of nine Baltic coastal lakes.

Samples were collected from 9 Baltic coastal ecosystems (Figure 1), varying in the level of hydrological connectivity, which was reflected in their salinity gradient. In the present study, all major lakes of the southern Baltic coast were sampled. This supplemented the information on the taxonomic composition of the benthic fauna obtained in the 2014–2015 study [17,21].

Lakes Łebsko, Resko, and Ptasi Raj are permanently connected with the sea (brackish), but Łebsko is the largest water body on the southern coast of the Baltic (71 ha), and Ptasi Raj is the smallest (<0.5 ha). Łebsko is fed by the Łeba River, while Resko by the Błotnica, both of which later flow into the sea. Lake Ptasi Raj is in fact a bay separated from the sea by a levee with a system of floodgates, which allow seawater intrusion. Lakes Liwia Łuża, Kopań, and Gardno (transitional: freshwater-brackish) are like estuaries, as they are mostly fed with fresh water but periodically are exposed to seawater intrusion. Lakes of this type are supplied with fresh water from an extensive system of drainage ditches (Liwia and Kopań) or a large river (Gardno). They also have a permanent connection with the sea (lakes Liwia and Gardno) or only a periodical one (Kopań). Freshwater lakes are represented by Wicko, Dołgie, and Sarbsko, devoid of hydrological connection with the sea. All the lakes receive pollution loads from the catchment, which accelerate their eutrophication [16,28]. 

Samples were collected primarily from soft lake sediments in 2019 and 2020 seasonally: in spring, summer, and autumn. Numbers of sampling sites depended on lake size: 4 in Ptasi Raj, 5 in Liwia Łuża, Resko, Kopań, Sarbsko, Gardno, and Dołgie, 8 in Wicko, and 11 in Łebsko. In each season at each site, 3 replicates of benthos samples were taken using an Ekman bottom dredge (0.03 m^2^ of the bottom each). The samples were sieved through a 0.5-mm mesh and next preserved with 6% formalin. In the laboratory, the bottom macroinvertebrates were sorted, identified to species level (if possible), and counted. We divided the organisms into 3 groups: opportunistic, euryhaline, and marine, as suggested by Reizopoulou et al. [23]. Opportunistic species are characterized by a low level of specialization and adapt to changes easily, while euryhaline species tolerate various levels of salinity. Identification and classification was based on available keys and information extracted from online databases, [29,30]. On the basis of biological data, α-diversity was assessed (Shannon index, H’).

Simultaneously with the biological sample collection, we measured physicochemical parameters at the same sites (in situ): salinity, dissolved oxygen (%DO), chlorophyll a concentration (Chl-a), NO_3_^−^, NO_2_^−^, and NH_4_^+^ with a calibrated AP-7000 Aquaprobe (AquaRead, UK). To determine total inorganic nitrogen (TIN), we summed up values of NO_3_^−^, NO_2_^−^, and NH_4_^+^ [31]. We also took water samples for laboratory analyses, including total phosphorus (TP). Laboratory analyses followed the Standard Methods [32]. Conductivity values (µS cm^−1^) were related to salinity values (PSU) as reported in Wagner et al. [33].

Differences between the three lake types in environmental parameters were assessed using principal component analysis (PCA). Spearman rank correlations (r) between biotic and abiotic parameters were calculated. Community structure was described using multidimensional scaling (MDS) based on a similarity matrix constructed using Bray–Curtis similarity index. Before the analysis, data for seasons from 2 years prior were averaged and log-transformed (y = log (x + 1)). Differences between variables for lake types were tested by analysis of variance (ANOVA) with the Kruskal–Wallis test by ranks (*p* < 0.05). ANOSIM test (R) was used for matrices describing the zoobenthos (numbers of opportunistic, euryhaline, and marine species), testing the null hypothesis that they did not differ significantly between the study lakes and seasons. Statistical analyses were performed using PRIMER v7 software.

## 3. Results

Environmental parameters varied widely between the lakes and seasons of sample collection (Table S1). Generally, brackish lakes had high temporal ranges of abiotic variables in spring and autumn, whereas freshwater ones had them in summer. Salinity gradients in brackish lakes were strongly sloping spatially, while transitional lakes more clearly varied seasonally.

Regardless of the season, the most important physicochemical parameters differentiating the abiotic conditions in the investigated coastal lakes were: salinity, conductivity, oxygen saturation and ammonium concentrations (one-way ANOVA, *p* < 0.0001). In addition, statistically significant differences in total phosphorus and total inorganic nitrogen were found in the seasons. Among the analyzed parameters, only the concentrations of chlorophyll remained similar (did not differ significantly statistically) in the studied lakes regardless of the season (Table 1).

**Figure 2:**
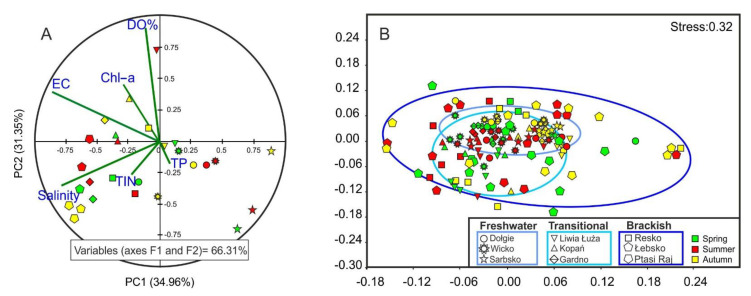
(**A**) PCA ordination on environmental parameters (salinity, EC, dissolved oxygen, Chl-a, TP and TIN) and (**B**) multidimensional scaling (MDS) plot based on species abundance.

Differences in variation of abiotic parameters in the study areas are probably due mostly to the level of primary productivity, aerobic conditions, and hydrological connection with the sea. The variation between the nine lakes (representing three lake types) in environmental parameters is illustrated by results of the PCA (Figure 2A). The PC1 axis (explains 35% of total variance) is associated with increasing salinity (and EC) gradient from low salinity in freshwater Lake Sarbsko in the right-hand part of the plot to sites with high salinity in the left-hand part of the plot (Lakes Ptasi Raj and Łebsko). Interestigly, it shows summer and autum effects of marine water intrusion to the lakes. The PC2 axis (31.35% of total variance) correlates positively with the aeration conditions in the lakes, while negatively with mineral nitrogen and phosphorus.

Multidimensional scaling based on qualitative-quantitative analysis of benthic fauna allowed us to identify benthic communities corresponding to individual types of coastal lakes. Seasonal variation was more conspicuous in brackish lakes, whereas in freshwater ones (with negligible temporal variation in salinity) community structure varied only slightly (Figure 2B). Species matrices for the whole data set can be classified into three major groups: euryhaline (tolerating a broad range of salinity levels and temperatures), opportunistic, and marine species. 

Among the analyzed physico-chemical parameters of water, the strongest correlations (Spearman’s ranks) with the abundance of benthic fauna in coastal lakes were found in concentrations of nitrates (r = 0.91), oxygen saturation (r = 0.77), and salinity (r = 0.66). The number of identified species (r = 0.93 and r = 0.76, respectively) and their α-diversity (r = 0.88 and r = 0.67, respectively) were clearly related to the concentration of TIN and NO_2_^-^. Benthic diversity was strongly associated with TP concentrations (r = 0.83).

In total, we identified 48 taxa of benthic invertebrates (of 10 taxonomic groups), including 26 opportunistic, 13 euryhaline, 7 marine, and 2 unclassified ones (Table 2). Because of the low salinity of the investigated lakes, their invertebrate community structure is unique, dominated by opportunistic species (= eurybionts) accompanied by small numbers of marine and euryhaline species. The level of hydrological connectivity between the sea and coastal lakes increased the taxonomic composition of benthic fauna groups. The species denisty in individual lakes is presented in Table S2. 

**Figure 3:**
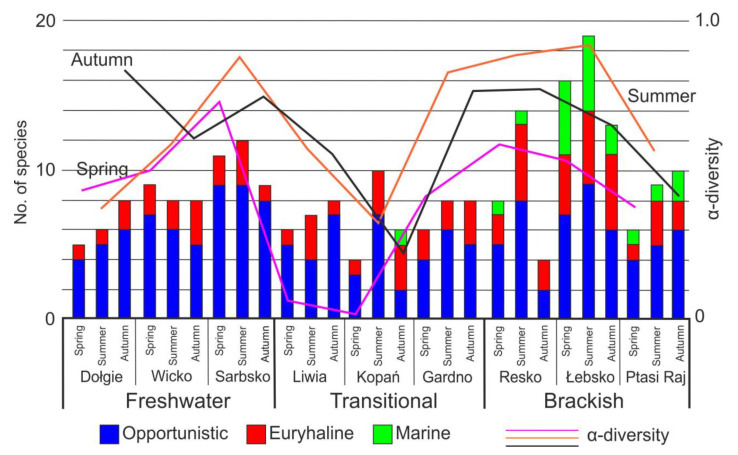
Richness (no of species) and α-diversity of species for each lake type in seasons.

The ANOSIM test of the species composition matrix showed that the lake types differed significantly at *p* < 0.001 except for transitional vs. freshwater, where significance level reached only 0.1. The greatest significance of differences was recorded for transitional vs. brackish lakes (R = 0.14, *p* < 0.001). The qualitative structure of benthic fauna did not differ significantly between seasons within lake types, but differences between lake types were noticeable. The strongest seasonal influence was observed between brackish and transitional lakes: in summer (R = 0.16, *p* < 0.01), spring (R = 0.16, *p* < 0.02), and autumn (R = 0.1, *p* < 0.05). Additionally, community structure in autumn differed between brackish and freshwater lakes (R = 0.12, *p* = 0.01). In lakes with higher salinity, communities “moved” between opportunistic and tolerant euryhaline species, depending on seasonality of changes in environmental parameters and intensity of seawater intrusion and eutrophication. However, opportunistic species were the major group of benthic fauna in the study lakes, accounting for 62% of the total number of identified taxa. The most common members of this group were *Chironomus f.l. plumosus* and *Polypedilum nubeculosum*. In brackish lakes with greater volume and undisturbed intrusion of seawater, marine and euryhaline species were more numerous (Figure 3). Overall, in brackish lakes, a greater total number of species was recorded and the resultant higher diversity was linked with seawater intrusion, reflected in the presence of marine species. The other lakes, with significant or total loss of hydrological connection with the sea, were characterized by lower diversity. In this context, transitional lakes were distinct, as in spring their benthic species diversity was the lowest.

In lakes of all types, the abundance of opportunistic invertebrates was to a large extent shaped by *C. plumosus*, which in spring reached up to 40% of the total catch. Their abundance was similar in brackish and transitional lakes, where they accounted for about 35% of the total benthic fauna. Euryhaline species reached the highest densities in brackish lakes in spring, in transitional ones in summer, and in freshwater ones in autumn. This group was represented most abundantly by *Gammarus duebeni* in transitional and brackish lakes, while by *Bezzia nobilis* in freshwater ones. Densities of marine organisms were high only in brackish lakes in summer. In lakes permanently connected with the sea, the most abundant marine species was *Hydrobia ulvae*, whereas in periodically connected ones, *Gammarus oceanicus*. 

**Figure 4:**
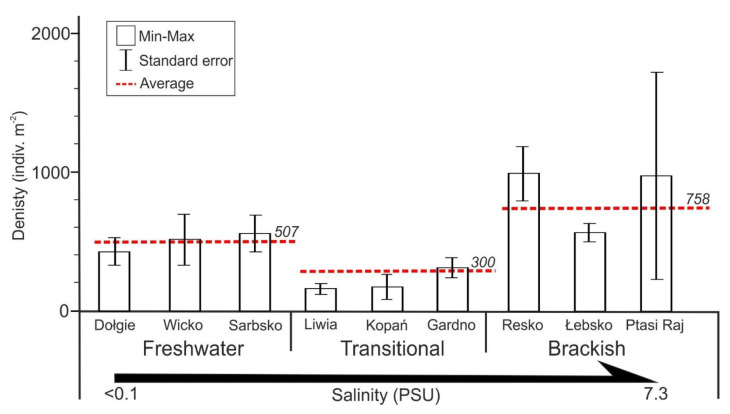
Density (10^3^ indiv. m^−2^) of benthic fauna for each lake. Bar chart (± standard error of mean) and dashed red line—average density for a lake type.

Benthic fauna reached the highest density in a brackish lake (Resko) and was the lowest in a transitional one (Kopań) (Figure 4). The abundance of benthic organisms was the highest in brackish costal lakes ($\bar{x}$ = 760 indiv. m^−2^), where the marine component of fauna were identified. Due to the greatest instability of environmental conditions, the lowest abundance ($\bar{x}$ = 300 indiv. m^−2^) was found in lakes periodically linked with the sea (transitional). Significant differences in density were found between brackish and transitional lakes (R = 0.060, *p* = 0.027) and freshwater and transitional lakes (R = 0.053, *p* = 0.029). Opposing salinity regimes (freshwater vs. brackish water) resulted in similar benthic fauna density values. 

In individual lake types, significant seasonal differences in the abundance of benthic fauna were observed only between spring and autumn, within all groups: brackish (R = 0.070, *p* = 0.024), transitional (R = 0.11, *p* = 0.003), and freshwater (R = 0.12, *p* = 0.003). Opportunistic species reached the highest densities in brackish lakes in autumn, in transitional lakes in spring, and in freshwater lakes in summer.

**Figure 5:**
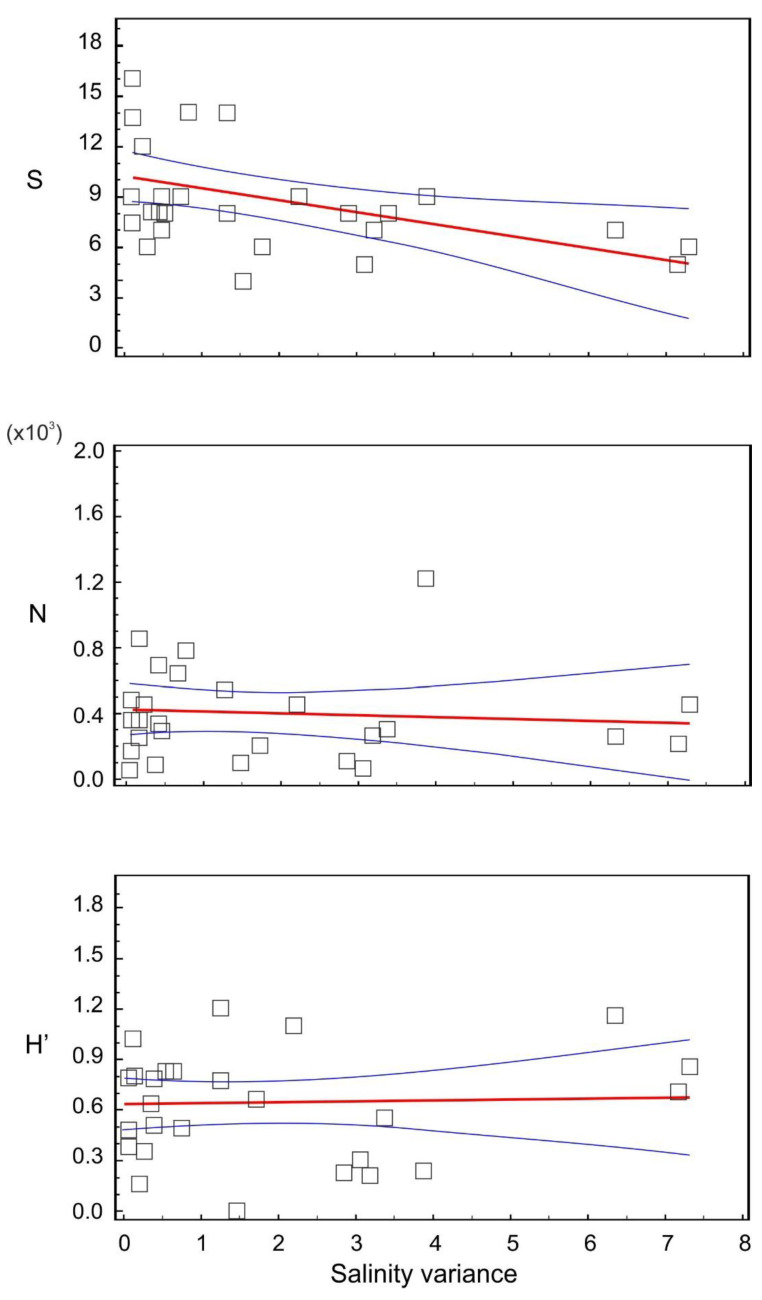
Mean diversity (H’), mean number of species (S) and mean abundance (N) versus salinity variance over the sampling occasions, blue lines indicate 95% confidence interval.

The overall data analysis (Figure 5) confirmed a high significance of the patterns associated with the level of environmental changes mean species richness (R = 0.37, *p* = 0.0001) and mean density (R = 0.10, *p* = 0.001) was negatively correlated with the variation in salinity, expressed as standard deviation of salinity values within the study lakes for each sample. In the case of α-diversity, salinity gradient also strongly affected the variation in benthic animal communities (R = 0.55, *p* = 0.0001). However, correlations between other benthos descriptors and mean salinity values were not statistically significant.

## 4. Discussion

Contributions of various predictors to shaping the natural gradients of water conditions in coastal water bodies depend on the major source of hydrodynamic energy in the system [1] and the intensity of intrusion of seawater [34]. Chemical and physical gradients affect animal and plant communities in many ways [35,36,37], so species richness in coastal lakes depends not only on salinity but on a complex of factors reflecting the degree of their isolation (time needed to restore marine conditions) [20,38]. When the time is longer, a stronger stimulus is needed to change the regime [17]. 

In coastal water bodies, the salinity gradient is the major environmental factor shaping species distribution [21,39,40,41,42,43]. It is particularly significant in the case of lakes connected with the Baltic Sea, which are brackish (~7 PSU). Coastal ecosystems with spatial variation in values of predictors were treated as ecotones and a two-ecocline model was proposed (physical transitional zone), which in the case of freshwater species is combined with gradients caused by the influx of fresh water (rivers, canals) to mid-estuary, and in the case of marine species, from the connection with the sea to mid-estuary [34,40]. In coastal water bodies, the environmental gradient is shaped mostly by seawater intrusion, strongly determined by the opposition of marine and terrestrial factors, and species richness following the one-scale pattern. In this study, the influence of seawater was the main factor determining the complexity of benthic fauna structure in the ecosystems being studied, while the degree of hydrological connectivity of each water body was optimally reflected in salinity and dissolved oxygen levels (Table 1). This concept has recently been the subject of numerous studies and has been well explained [42]. Differences in natural stress were also reflected in the presence of various invertebrate groups. In the communities being studied, opportunistic species formed the major group, including common species of shallow eutrophic lowland lakes (e.g., *C. plumosus*). They are highly tolerant to osmotic stress, which allows them to colonize abundantly coastal lakes [24]. They are accompanied by euryhaline species, treated as characteristic of habitats of this type. Marine species were limited to brackish lakes (Łebsko, Resko, and Ptasi Raj), where they increased the species richness of the ecosystem (see Table 2). An increasing number of reports emphasize the ecological significance of the dependence between seawater intrusion within systems and biotic zonation [21,23,44,45]. In the Baltic coastal lakes under study, we observed a positive linear relationship between species richness (S) and the slope of the salinity gradient in each water body, which indicates their dependence on seawater intrusion. The higher the salinity gradient, the lower the mean number of species in individual ecosystems. Benthic fauna abundance (S) and diversity (H’) showed a similar pattern, but the correlation with salinity variance was feeble. It seems that the abundance is more strongly linked with food availability and seasonal biological cycles, while species richness is mostly connected with the intensity of environmental stress. In a similar study of three Mediterranean lagoons with a broader salinity gradient (0–12 PSU), stronger positive correlations of species richness and diversity with salinity and weaker correlations in the case of abundance were observed [23]. 

Seasons seem to less strongly affect the structure of brackish communities, which was confirmed by MDS results (Figure 2B). Similar results were reported by Obolewski et al. [17] who stated that temporal changes in species richness and community structure often prove to be non-significant in coastal water bodies, irrespective of their hydroecological type. According to Nicolaidou [46], the lack of seasonality is attributed mostly to continuous reproduction of some abundant species and both trophic and other interactions between species. It is worth noting that only our most recent studies provide sufficient information on the level of taxonomic diversity of benthic invertebrates. Therefore, the presented results can only be treated as fully representative for the coastal lakes of the southern Baltic Sea. Compared to the results obtained from our research from 2014–2015, we managed to identify a much larger number of species. In these studies, 48 taxa were found, while only 28 were found in the earlier one [17].

The high trophic state of Baltic coastal lakes was reported many times [28,47,48]. Taking into account the specificity of saline conditions, the coastal habitats with a small number of species and low diversity should be carefully assessed as severely degraded. In particular, in the case of transitional coastal lakes, low biodiversity rates are due to natural stress caused by: (i) unstable salinity conditions that benthic species must tolerate to survive and (ii) variable environmental parameters due to seawater intrusion. Our observations showed that there is a specific dispersion of benthic fauna in the gradient zone between sea and land along which the intensity of salinity stress varies from mild to severe. It is worth noting that salinity, controlled by the hydrodynamics of water at the interface between the lake and sea, influences the variability of the physicochemical conditions of the coastal environments. In light of the above, our study points to the role of brackish sea water intrusion as a factor in improving oxygenation in the bottom zones of lakes. It is worth emphasizing that in the case of coastal lakes, diffusion and photosynthesis, as well as the inflow of oxygenated sea waters are responsible for the oxygenation of the waters [18,49].

The measures of diversity and the associated biotic indices proved to be less effective for coastal water bodies with a narrow salinity gradient. This was mostly due to the dominance of species tolerant of natural stress factors [17,18,27,49,50]. This situation was described as a “paradox of estuary quality” and can lead to false classifications based on the level of hydrological connectivity [51,52]. The occurrence of intrusion of brackish seawater causes “refreshment” of the biotope but destabilizes environmental conditions [16,53]. Moreover, hydrological connectivity also constitutes a “window of opportunity” for migration of alien marine and euryhaline species, and their expansion from the Baltic Sea to lakes [54,55].

## 5. Conclusions

Salinity is a key determinant of species diversity in coastal waters. Species richness and diversity of benthic communities in coastal waters are strongly linked with environmental gradients determined by the level of hydrological connection with the sea. Various indices used to assess the ecological potential of water bodies, including diversity indices, should take into account the level of natural instability evoked by marine water intrusions. This aspect concerns both the potential chances of migration and adaptation of organisms, but also leads to permanent changes in habitat conditions and dominance of opportunistic species. Assessment of biodiversity along salinity gradients in coastal lakes is the only and optimal way to recognize natural or anthropogenic stress factors. Salinity should be a prerequisite for the proper assessment of ecological status and the development of effective methods of protecting coastal lakes.

## Figures and Tables

**Table 1:** Physico-chemical characteristics of water in Baltic coastal lakes. Values are means of samplings (2019–2020) grouped for seasons ± standard deviations and results of one-way ANOVA evaluating differences in results (Kruskal–Wallis test, significance level when *p* < 0.05). The specific conductivity (EC) value was related to salinity according to Wagner et al. [33].

		Freshwater			Transitional			Brackish			*p*
		Dołgie	Wicko	Sarbsko	Liwia	Kopań	Gardno	Resko	Łebsko	Ptasi Raj	
Spring	Salinity (PSU)	0.02 ± 0.00	0.07 ± 0.01	0.23 ± 0.04	0.47 ± 0.07	0.77 ± 0.08	0.81 ± 0.36	1.48 ± 0.43	2.20 ± 0.72	6.31 ± 0.54	<0.0001
	EC (µS cm^−1^)	47 ± 2	151 ± 17	483 ± 86	988 ± 144	1576 ± 154	1649 ± 735	2873 ± 819	4129 ± 1285	11088 ± 847	<0.0001
	O_2_ (%)	96.3 ± 9.0	111.2 ± 14.8	80.5 ± 14.5	113.3 ± 10.3	112.4 ± 12.7	87.4 ± 9.9	96.1 ± 31.4	90.1 ± 19.2	109.3 ± 37.7	<0.0001
	Chl-a (mg L^−1^)	21.1 ± 22.4	23.9 ± 24.5	25.9 ± 27.0	17.1 ± 19.1	25.7 ± 21.3	18.4 ± 18.5	14.0 ± 15.8	13.3 ± 12.2	14.7 ± 9.2	0.81
	TP (mg L^−1^)	0.32 ± 0.06	0.57 ± 0.23	0.32 ± 0.28	0.71 ± 0.37	0.43 ± 0.13	0.19 ± 0.05	0.36 ± 0.15	0.30 ± 0.12	0.50 ± 0.38	<0.0001
	NO_3_^−^ (mg L^−1^)	0.56 ± 0.30	0.86 ± 0.46	0.98 ± 1.04	1.35 ± 1.77	0.90 ± 0.44	0.58 ± 0.24	0.98 ± 0.61	0.97 ± 0.49	1.91 ± 1.82	0.03
	NO_2_^−^ (mg L^−1^)	0.21 ± 0.17	0.52 ± 0.27	0.64 ± 0.33	0.72 ± 0.31	0.61 ± 0.26	0.51 ± 0.24	0.57 ± 0.22	0.74 ± 0.85	0.77 ± 0.40	0.005
	NH_4_^+^ (mg L^−1^)	0.36 ± 0.28	0.25 ± 0.16	0.24 ± 0.07	0.18 ± 0.12	0.18 ± 0.11	0.27 ± 0.19	0.06 ± 0.15	0.36 ± 0.25	0.68 ± 0.42	<0.0001
	TIN (mg L^−1^)	1.14 ± 0.64	1.55 ± 0.83	1.86 ± 1.28	2.25 ± 1.96	1.68 ± 0.64	1.36 ± 0.41	1.60 ± 0.9	2.08 ± 0.89	3.36 ± 2.56	0.02
Summer	Salinity (PSU)	0.02 ± 0.00	0.13 ± 0.07	0.31 ± 0.24	0.54 ± 0.12	1.26 ± 0.23	1.29 ± 1.04	2.76 ± 1.14	2.80 ± 0.64	7.12 ± 0.45	<0.0001
	EC (µS cm^−1^)	50 ± 3	282 ± 139	630 ± 468	1107 ± 237	2560 ± 451	2535 ± 1938	5113 ± 1997	5204 ± 1109	12415 ± 758	<0.0001
	O_2_ (%)	107.5 ± 30.2	109.7 ± 21.3	95.8 ± 33.0	148.1 ± 30.1	111.0 ± 24.8	93.2 ± 20.1	90.3 ± 19.4	82.0 ± 18.8	83.9 ± 35.8	<0.0001
	Chl-a (mg L^−1^)	12.3 ± 7.3	18.6 ± 16.8	28.3 ± 25.5	16.8 ± 18.7	26.5 ± 24.1	17.3 ± 14.9	22.5 ± 2262	14.8 ± 16.3	12.0 ± 9.2	0.78
	TP (mg L^−1^)	0.33 ± 0.13	0.37 ± 0.28	0.35 ± 0.37	0.38 ± 0.22	0.32 ± 0.19	0.16 ± 0.12	0.42 ± 0.27	0.25 ± 0.12	0.62 ± 0.23	<0.002
	NO_3_^−^ (mg L^−1^)	0.56 ± 0.24	0.81 ± 0.48	0.79 ± 0.73	0.42 ± 0.20	0.87 ± 0.53	0.94 ± 0.44	0.99 ± 0.75	1.02 ± 0.61	1.40 ± 0.72	0.03
	NO_2_^−^ (mg L^−1^)	0.33 ± 0.19	0.53 ± 0.36	0.43 ± 0.32	0.34 ± 0.13	0.53 ± 0.26	0.56 ± 0.21	0.56 ± 0.24	0.83 ± 1.42	0.73 ± 0.38	0.19
	NH_4_^+^ (mg L^−1^)	0.31 ± 0.25	0.61 ± 1.18	0.27 ± 0.19	0.16 ± 0.20	0.21 ± 0.08	0.55 ± 0.46	0.01 ± 0.01	0.36 ± 0.20	0.57 ± 0.41	<0.0001
	TIN (mg L^−1^)	1.20 ± 0.55	2.07 ± 1.30	1.48 ± 0.91	0.93 ± 0.22	1.61 ± 0.73	2.05 ± 0.95	1.56 ± 0.79	2.20 ± 1.62	2.71 ± 1.46	0.005
Autumn	Salinity (PSU)	0.02 ± 0.00	0.15 ± 0.09	0.46 ± 0.20	0.57 ± 0.23	1.27 ± 0.29	1.33 ± 0.57	3.17 ± 1.43	2.95 ± 0.74	7.28 ± 0.16	<0.0001
	EC (µS cm^−1^)	50 ± 2	325 ± 182	921 ± 395	1156 ± 443	2522 ± 511	2642 ± 1111	5797 ± 2502	5455 ± 1284	12681 ± 280	<0.0001
	O_2_ (%)	106.6 ± 13.1	91.8 ± 8.9	117.6 ± 16.5	112.0 ± 16.7	127.5 ± 16.6	118.2 ± 9.1	117.9 ± 16.7	73.1 ± 34.8	75.1 ± 17.8	<0.0001
	Chl-a (mg L^−1^)	20.3 ± 17.8	20.4 ± 19.4	32.8 ± 34.6	16.6 ± 21.3	28.9 ± 25.1	20.1 ± 20.3	11.1 ± 9.0	12.7 ± 12.2	17.4 ± 11.7	0.60
	TP (mg L^−1^)	0.27 ± 0.12	0.46 ± 0.18	0.31 ± 0.28	0.40 ± 0.46	0.30 ± 0.20	0.14 ± 0.06	0.42 ± 0.22	0.30 ± 0.16	0.58 ± 0.27	0.0003
	NO_3_^−^ (mg L^−1^)	0.64 ± 0.39	0.82 ± 0.39	0.68 ± 0.80	0.86 ± 0.28	1.05 ± 0.61	1.61 ± 1.39	1.62 ± 0.99	0.74 ± 0.39	1.21 ± 0.60	<0.05
	NO_2_^−^ (mg L^−1^)	0.40 ± 0.22	0.49 ± 0.23	0.48 ± 0.55	0.58 ± 0.12	0.66 ± 0.23	0.60 ± 0.20	0.56 ± 0.21	0.58 ± 0.21	0.80 ± 0.50	0.30
	NH_4_^+^ (mg L^−1^)	0.34 ± 0.27	0.33 ± 0.27	0.24 ± 0.46	0.25 ± 0.35	0.14 ± 0.09	0.48 ± 0.48	0.05 ± 0.08	0.29 ± 0.15	0.53 ± 0.29	<0.0001
	TIN (mg L^−1^)	1.37 ± 0.57	1.60 ± 0.74	1.12 ± 1.20	1.70 ± 0.37	1.85 ± 0.75	2.70 ± 1.32	2.23 ± 1.21	1.61 ± 0.53	2.54 ± 1.35	<0.04

**Table 2:** The composition of species for each study area (+ the presence of a taxon). GS = Groups of species: O-Opportunistic; E-Euryhaline; M-Marine. Classification according to [17,29,30].

	GS	Freshwater			Transitional			Brackish		
		Dołgie	Wicko	Sarbsko	Liwia	Kopań	Gardno	Resko	Łebsko	Ptasi Raj
Oligochaeta		+	+	+	+	+	+	+	+	+
Polychaeta										
*Hediste diversicolor*	M								+	+
*Pygospio elegans*	M								+	
Crustacea										
*Asellus aquaticus*	O				+			+	+	
*Gammarus duebeni*	E				+	+	+	+	+	+
*Gammarus oceanicus*	M					+		+	+	+
*Corophium volutator*	E							+		
*Praunus flexuosus*	E								+	
*Idotea balthica*	M							+		
*Neomysis integer*	M								+	
*Palaemon elegans*	E									+
Hirudinea										
*Glossiphonia complanata*	O				+	+				
*Erpobdella octoculata*	O				+			+		
*Piscicola geometra*	E								+	
Diptera larvae										
*Chironomus* f.l. *plumosus*	O	+	+	+	+	+	+	+	+	+
*Chironomus* f.l. *thummi*	E								+	
*Glyptotendipes* e.g., *gripekoveni*	O		+					+		+
*Procladius* spp.	O	+	+	+	+	+	+	+	+	+
*Polypedilum nubeculosum*	O	+	+	+	+	+	+	+	+	+
*Polypedilum* e.g., *scalaeum*	O		+	+		+			+	+
*Psectrocladius barbimanus*	E	+	+	+	+	+	+	+	+	+
*Bezzia nobilis*	E	+	+	+	+		+	+		+
*Microtendipes* e.g., *chloris*	O							+	+	
*Sergentia coracina*	O	+	+	+	+	+	+	+	+	+
*Einfeldia* e.g., *carbonaria*	O		+					+	+	
*Clunio* sp.	M								+	
*Chaoborus* sp.	O	+								
*Diamesa campestris*	E		+				+			+
*Tanatyrus* e.g., *mancus*	O					+				
Chironomidae n.det.		+	+	+	+		+	+	+	
Hemiptera										
*Sigara* sp.	O				+					
*Corixa* sp.	O							+		
Trichoptera larvae										
*Triaenodes bicolor*	E				+					
*Limnephilus* sp.	O					+				
*Limnephilus bipunctatus*	O	+						+		
*Ecnomus tenellus*	O	+		+						
Ephemeroptera larvae										
*Caenis macrura*	O	+								
Gastropoda										
*Bithynia tentaculata*	O			+	+	+		+	+	+
*Planorbis planorbis*	O				+					
*Valvata piscinalis*	O		+	+					+	
*Theodoxus fluviatilis*	E		+	+		+			+	
*Potamopyrgus jenkinsi*	E								+	+
*Hydrobia ulvae*	M								+	+
Bivalvia										
*Dreissena polymorpha*	E		+	+		+		+	+	+
*Pisidium* sp.	O		+	+						
*Unio tumidus*	O			+			+			
*Unio pictorum*	O		+	+						
*Anodonta anatina*	O			+			+		+	
Number of taxa		12	17	18	16	15	12	21	27	18
Number of taxa in type of lakes		24			25			36		

## Data Availability

All source data can be found in the attached supplementary materials.

## Supplementary Materials

The following is available online at https://www.mdpi.com/article/10.3390/ani11113039/s1, Table S1: Physicochemical properties of water in coastal lakes of the Baltic Sea during the study period 2019–2020, Table S2: Composition and mean abundances of macroinvertebrates (indiv. m^−2^) at the coastal lakes of the Baltic Sea during the study period 2019–2020. Classification into three groups of organisms: M- marine; E-euryhaline; O-opportunistic.
